# What functions do palliative care bereavement services deliver? A scoping review

**DOI:** 10.1177/26323524251326947

**Published:** 2025-03-22

**Authors:** Kathleen E. Jurgens, David C. Currow, Jennifer Tieman

**Affiliations:** Research Centre for Palliative Care, Death and Dying (RePaDD), College of Nursing and Health Sciences, Flinders University, SA 5001, Australia; Faculty of Health, University of Technology Sydney, Sydney, Australia; Research Centre for Palliative Care, Death and Dying (RePaDD), College of Nursing and Health Sciences, Flinders University, SA, Australia

**Keywords:** bereavement, bereavement services, function, interventions, palliative care, scoping review, support

## Abstract

Following someone’s death, bereaved people may struggle with their grief. When a patient receives palliative care, bereavement support for the patient’s family is an expected function of specialist palliative care services. To date, detailed descriptions of the purpose, function and provision of support from bereavement services are limited. This study examined how specialist palliative bereavement services self-defined their functions and described any support and interventions they offer. The aim was to synthesise how services satisfied their responsibilities for continuity of support to the family following a patient dying. A scoping review was undertaken to examine the literature on bereavement support within palliative care services to codify how they initiated post-death contact, the purpose of contact and what interventions were offered. Seven databases were interrogated in 2020 using search terms developed by CareSearch (www.caresearch.com.au) with refinement. Items needed to be in English and detail the aims and functions of service-initiated contacts and support. Information was thematically analysed using an inductive approach. Bereavement contact from palliative care services had an overall aim of offering guidance through the provision of information, access to a risk assessment and counselling. The analysis demonstrated the provision of bereavement information, describing support pathways and delivery of accessible grief interventions provided a ‘safety net’. Other themes revealed services often monitored adjustment through scheduled reviews, were aware of their limitations and completed referrals to other services as needed. This study adds to our understanding of palliative care bereavement services and provides valuable information about their intended functions. To improve understanding of bereavement functions, services need to clearly define their primary purpose and how this meets the needs of bereaved people and national standards. Ideally, future research would interview bereavement staff directly to ensure accurate descriptions of service aims and model.

## Plain language summary

A study on the aims of palliative bereavement service contact

Bereavement care and contact is an expected component of specialist palliative services and is written into standards and guidelines. The purpose of service-led contact is not clear and there is some suggestion that it is unnecessary. While not every person bereaved from an anticipated death will require formal grief intervention, around 10% of bereaved people a higher level of morbidity, mortality and/or prolonged grief. Few studies have identified the aim of service-led bereavement contact. Our review of the literature identifies they have a common purpose. Thematic analysis showed that service-led contact aims to act as a safety net bereaved individuals following an anticipated death. This study demonstrates the importance of bereavement support from specialist palliative service. Contact provides an important support of people who need more information about grief and/or formal interventions. However, greater consistency in describing the purpose of service-led bereavement support is needed. Further research is required to examine current models of service-led contact from palliative bereavement teams to identify how they align with standards and expectations of care.

## Introduction

The World Health Organization describes palliative care as an approach that improves patients quality of life, respects choices, and supports patients families during the illness and into bereavement.^1^ In keeping with the World Health Organization’s definition, Palliative Care Australia has developed standards that include the expectation to support the patient and their family.^2^

Evidence reflects that service-initiated contact with bereaved caregivers is a standard practice in many services, written into guidelines, and generally provided through a paid position within palliative care services.^3–7^ Services typically provide contact using telephone, in-person or mailed information.^7–10^ Service-led contact is characterised as sharing information, offering a review of coping and provision of additional grief interventions where available.^3,4,6–8^ The case for contacting newly bereaved caregivers seeks to be justified given the concerns regarding the risk of increased morbidity including depression, anxiety, prolonged grief and increased rates of mortality.^11–19^ Negative changes in health or an exacerbation of existing health concerns may be experienced for several months following the death of someone significant. Health-related bereavement impacts may include major depression, anxiety, post-traumatic stress disorder, substance abuse, suicide risk, heart conditions and gastrointestinal disorders as well as poor sleep. Similarly, research recognises there may be negative financial and practical impacts through bereavement, including access to superannuation or funeral payments and loss of an income.^19,20^ Information to carers and families should facilitate knowledge of typical grief reactions and where to access support if required.^21^ The intention of service-led contact is to provide information (grief literacy) and, for a smaller proportion, grief counselling. Such support potentially aids in identifying and supporting those at risk of struggling to adjust. However, there is little or no evidence available that such contacts and interventions lead to better outcomes for people.

It has been argued that universal service-led contact leads to unnecessary interventions.^22–27^ Examples of references to unnecessary interventions ‘ineffective outreach’ where bereaved individuals are ‘offered help without them asking for it’^28^ or that ‘most palliative care services tend to provide blanket interventions . . . regardless of (people’s) support needs’.^25^ In contrast, other research notes the difference between bereavement services providing ‘*access*’ to support and the bereaved person’s engagement in grief counselling interventions.^6^ Claims about unnecessary interventions may lead to a reduction of bereavement support availability, as specialist palliative services look to rationalise staff time to meet broader service demands. Other studies report that not all bereaved people who are aware of and eligible to receive support use it. Therefore, concerns regarding unnecessary offers of assistance may not be warranted.^10,29,30^ It has also been noted that bereaved people may not seek out bereavement support directly.^31^ If service-initiated bereavement contact does not occur, it is unclear how those who are grieving will locate information about available services or receive guidance on when to seek support – especially as bereaved people can become disconnected from health and care services.^32^ People experiencing bereavement have expressed such concerns themselves through not knowing how to access desired support or describe discomfort in asking for help.^10,33,34^ Bereaved caregivers may also report a sense of feeling abandoned following the patient’s death, especially when care had been intensive or provided over long periods of time.^35,36^ When seeking information about how bereavement services are structured and function, detailed descriptions of services are difficult to locate. Where information is available (typically through surveys of palliative care services), even then, it often lacks details about service models and functions.^37^ Understanding both the intention and practice of palliative bereavement services models are necessary given an increasing rate of deaths and service funding pressures, which could make it difficult to fulfil specified standards of bereavement care.

## Research objectives

While surveys identify the general characteristics of adult palliative bereavement services, clarity about their aims and purposes was lacking. To bring the literature together, we conducted a scoping review to examine adult palliative care services’ self-described approaches to service-initiated bereavement contacts and intervention practices following a patient’s death. There was a specific focus on how the services described the aim of their contact and any subsequent interventions.

## Materials and methods

A scoping review was selected to undertake a comprehensive examination of how adult palliative bereavement services described their models. The approach was chosen following recommendations.^38–40^ A scoping review is ideally suited when exploring a topic when the available evidence is unclear.^39^ Scoping reviews are a systematic search for information through a transparent and reproducible method.^39^ Ensuring that a clear protocol is followed that reduces error and extracts and presents data in a structured manner will increase reliability. For a standardised and reliable scoping study process, a five-stage methodology is suggested where research questions are identified; relevant studies are identified and selected; data are charted; and results are gathered, analysed and reported.^40^ The PRISMA Extension for Scoping Reviews (PRISMA-ScR) template was used as a guide in planning, conducting and reporting the study, though the review was not registered (Supplemental Material 1).^41^ Our emphasis was placed on developing a clearly written, comprehensive research protocol. Critical features of our protocol included defining the study rationale, eligibility criteria, information sources, data charting processes and summarising evidence. Scoping studies have the capacity to increase the sensitive review of information through an iterative process as the study completes the analysis of the literature.^39–41^ To facilitate a thorough examination of the information, an extraction template was developed to consistently record details regarding the described function and method of service-led bereavement contact and approach to grief interventions. The scoping review was directly influenced by the primary researcher’s clinical experience in adult palliative care services. This led to limiting the review to specific information that was identified as difficult to obtain and the potential impacts of this gap. It is recommended that researchers recognise and discuss how the research and data examined influence one another, both prospectively regarding decisions made at the outset of the study and retrospectively when writing the results. This process is referred to as reflexivity.^42^ Responding to claims about unnecessary interventions being undertaken, we reviewed the literature that described bereavement services in specialist palliative or hospice services for adults with life-limiting illnesses. In deciding to focus on adult-only services, the primary researcher considered her clinical experience in adult specialist palliative care. An earlier unpublished literature review by the primary researcher highlighted the need to locate evidence regarding the aims and functions of bereavement services in adult palliative care settings.^43^ Therefore, we excluded information about paediatric services. We also limited the review to information on the specific actions undertaken by bereavement services, rather than any condolence actions completed by the wider palliative care or hospice team for the same reason. To enhance the rigour of this study, two independent co-reviewers (a Clinical Psychologist and a PhD qualified Social Worker) supported the primary researcher in the initial screening of abstracts and titles, full review of the included articles, and thematic analysis. Literature was included if it provided sufficient details regarding palliative bereavement service-initiated contact with an identified person from the patient’s medical record after they died.

### Search strategy and eligibility criteria methodology

Search terms for the review were initially identified by examining searches used within the Australian palliative care knowledge database, CareSearch (Caresearch.com.au), to identify relevant literature and evidence. CareSearch is a specialised online database of palliative care and its associated disciplines.^44^ Further refinement was undertaken to identify descriptions of service-initiated bereavement contact from palliative or hospice services themselves (Supplemental Material 2). A specialist university research librarian was consulted and worked alongside the primary researcher to ensure the best results. We jointly worked through our existing knowledge, use of database thesauruses and joint brainstorming of useful search terms. Search terms were refined through trial searches until the expected literature was retrieved. A systematic search was conducted on 29 February 2020 (rerun with original date parameters on 31st December 2024) using seven databases. CINAHL, Cochrane, PsychINFO, Web of Science, Scopus, ProQuest and MEDLINE databases were used. Additional visual scanning of the reference lists from the relevant studies was also performed. The results were screened based on inclusion and exclusion criteria which are provided below in Table 1.

**Table 1.** Inclusion and exclusion criteria.

Inclusion criteria	Exclusion – out of scope criteria
English language	Paediatric palliative/hospice services
Description of service-initiated contact to bereaved	Community nursing services that were not employed by palliative services
Adult palliative bereavement setting	Testing of specialised bereavement interventions
Anytime to 1 March 2020	Future service development/recommendations
Grey literature (theses, service reports, etc.)	Condolence, anniversary or other contacts made by palliative care nursing or medical staff as this is independent of the bereavement service
	Opinion pieces, abstracts, posters
	Where limited information was provided about the purpose and characteristics specific to the bereavement support model, such as memorial services, anniversary mailings or case studies

**Figure 1.**
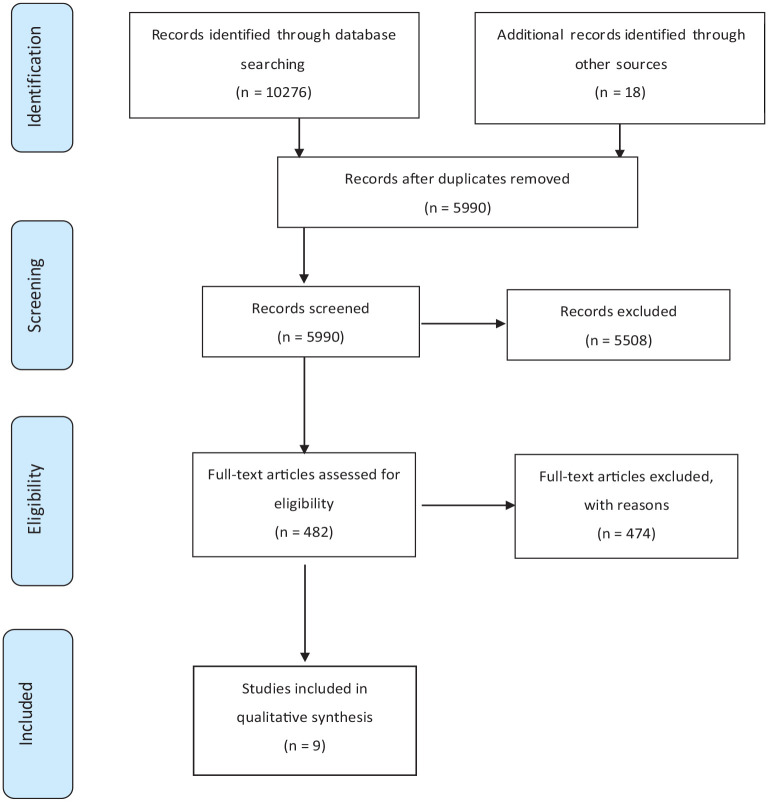
PRISMA flow diagram of search results.

The database search identified 10,276 articles and grey literature, which were then imported into Covidence. Covidence is a web-based collaboration software platform that streamlines the production of systematic and other literature reviews (Covidence systematic review software; Veritas Health Innovation, Melbourne, VIC, Australia). A PRISMA flow diagram of the screening process is shown in Figure 1. Duplicates were removed, leaving 5990 records. The primary researcher screened titles and scanned abstracts, checking the articles against the exclusion/inclusion criteria. Co-reviewers randomly checked 210 (3.5%) articles at this stage, reviewing both the excluded ones and those to be screened in more detail. A 97% agreement rate was achieved for title and abstract screening, with conflicts being resolved through consensus. Next, the primary researcher thoroughly reviewed the abstracts of 482 full-text articles for descriptions of service-initiated bereavement contact. Survey reports regarding palliative bereavement services were excluded. This decision was based on reporting typical features of service models where the research did not clarify the aim or function of interviewing bereavement staff. Texts focussed solely on describing the process of bereavement contact through sending anniversary cards or condolence notes or that failed to clearly describe service-initiated contact of bereaved family were also excluded.

Initially, 26 articles had preliminary data extracted by the primary researcher. The results were then shared between the two reviewers for independent reading before the team discussion. The team review resulted in a significant refinement of the extraction focus, with 18 articles subsequently excluded. Articles were removed if they did not adequately describe the bereavement service contact or method. Consensus was sought again before the final decision regarding inclusion was made. Nine papers met the criteria for inclusion in the review, with the evidence summarised in Table 2. The data-extraction template is provided (Supplemental Material 3).

**Table 2.** Synthesis of articles regarding service-initiated contact, assessment aim and grief interventions.

References	Setting, method of article/study	Timing and method of service-initiated contact method	Purpose of service-initiated bereavement contact; delivery of interventions, overview of supports available and duration of service
Reid et al.^9^	Five hospices in the United Kingdom. Semi-structured interviews with staff and volunteers and bereaved people who had and had not accessed bereavement support	Unsolicited contact with bereaved people shortly after the patient had died to offer support	To ensure support was available, to make accessing support easier, and to facilitate making sense of and adjusting to the death. Noted that the intention of the grief counselling, when commenced, was described by some services as assisting family members to ‘normalise . . . clients’ grief and . . . accompanying the person on their journey’ (p. 432). Though not stated, services indicated that grief counselling would cease once the individual was able to ‘. . .live with their grief on their own’ (p. 432)
Lattanzi^45^	Single free-standing Hospice, Colorado, USA. Description of service model	Active outreach is undertaken initially by telephone. High-risk individuals seen within weeks of patient’s death through individual visits and telephone contacts	Supportive in approach, seeking to support, educate and link to community or private therapists as indicated. 80%–85% are provided formal contact (pp. 56–58). Individual support average of 3–4 contacts, with telephone calls and notes viewed as important (p. 59). Author articulated the need for clear goals and functions for bereavement services. Described grief support as an ‘. . . opportunity to review and reflect on the experience of. . .’ (p. 56) caregiving
Harter Janson^46^	Single site – Hospice unit located in a Rehabilitation Hospital, Colorado, USA. Service description as developed and administered by author	Bereaved individuals were regularly contacted from 2 weeks after the patient’s death. Clear delineation and handover process from hospice staff and hospice volunteers to bereavement service	Programme usually provided by bereavement volunteers and continued throughout first 13 months at set time points. Aim of programme were to offer guidance, provided educational materials, support expression of grief and enhance coping strategies. Services described as comprehensive and tailored to client’s changing needs and wishes. ‘Volunteers . . . encouraged to schedule a visit . . . during the first two weeks of bereavement’ (p. 131). Four forms of support available (individual, social group, therapeutic group and regular supportive correspondence)
Souter and Moore^47^	Single site, Canadian Palliative Care Unit with 36 beds situated within a Toronto hospital. Programme evaluation via review of case notes and posted evaluation survey to bereaved completing 12 months of support	A telephone call at 2 weeks with frequency of ongoing personal contact via telephone based upon initial assessment	Described as a 12-month service-initiated bereavement contact process to one person connected to the deceased patient, drawing on a volunteer workforce with the guidance of a Bereavement Coordinator. Bereavement contact was ‘. . . to offer reassurance and support’ (p. 34) to help facilitate ‘. . . the grieving process’ (p. 34) as the person gradually adjusted to their loss
Bolotin^48^	Single site – Faith-based not-for-profit Hospice, Washington, USA. Service description, with reference to feedback surveys from bereaved people	One or more family members contacted by telephone initially. Service expanded with an aim to complete telephone contact to one person for 100% patients within 30 days	To support families during the difficult transition. Described outreach as a process of ‘extending the benefits of hospice support during the year after death. . .’ (p. 47)
Agnew et al.^49^	Ten Marie Curie Hospices, UK. Qualitative study – telephone semi-structure interview of 10 bereavement service leads to review their current practice of bereavement assessment	Contact commenced between 5 and 10 days if considered to be at risk by telephone. Provision of information and support options. Written information provided within 12 weeks if low risk	Contact was made to offer information on bereavement support. Terms used to describe the process of service-initiated contact included to ‘. . .offer information and support. . .to minimise. . . risk of falling through the net’ (p. 20). The service models encouraged self-referral as a way of respecting self-determination and autonomy while promoting personal resilience
Eastman et al.^50^	Single service hospital-based palliative care unit, Melbourne, Australia. Brief description of implementation and use of bereavement programme	Active contact to those bereaved scored as moderate to high risk, who were telephoned at 3 weeks post-death	Supportive telephone contact for those deemed moderate to high risk. Contact provided for a short time following the patient’s death, though duration was not clear
Khumalo and Maasdorp^51^	Zimbabwe, Africa, The Island Hospice and Health Care Community Service. Service description	The families continue to be seen/visited throughout the first year following the patient’s death, with a focus on monitoring and assessing the children’s coping due to their vulnerability	The service expresses provision of a comprehensive bereavement service. ‘The family is seen during follow-up bereavement visits and particular timings . . . during the first 12 months. . . although this is not formulaic in its application’ (p. 4)
Ghesquiere et al.^52^	Single site – Hospice unit located in acute hospital, New York, USA. External review of de-identified electronic records of 3561 bereavement charts	Automatically followed up, initially by telephone to complete a bereavement assessment within 38 days of death for up to six individuals per patient	Bereavement assessment to discuss possible needs, with additional services able to be offered, including groups, family and individual counselling and regular telephone review

### Data charting and synthesis

A selective data-extraction process to meet our objectives was undertaken following Noyes and Lewin,^53^ who advised three central foundations are needed for the examination and extraction of qualitative evidence. First, the purpose of the review forms the basis for data gathering. Second, an extraction template is designed to match the purpose of the study. The final central foundation recommends that the research process is an iterative one. This involves a process of reading, data extraction and synthesis of information that is cyclic, allowing key themes to emerge. The primary researcher developed an extraction template to allow for the documentation of keywords and information. Content for the template followed Johanna Briggs Institute^38,41^ recommendations of participant, *c*oncept and *c*ontext, known as PCC. Study quality and bias analyses were not completed because the purpose of the study was to obtain reasonable descriptions of bereavement service contact and aim rather than to examine the quality of the article or interventions. The extraction template recorded the following information:

(a) Article identifiers (author, year, funding sources);
(b) Setting of bereavement support (participants and context);
(c) Method and purpose of service-initiated bereavement contacts; and
(d) Approach to assessment and grief interventions.

Additional information was extracted, although this has not been reported here.

### Process of thematic analysis and synthesis of evidence

The extracted information was thematically analysed using an inductive approach, to identify the purpose and nature of service-initiated contact in palliative bereavement services. A manual process was used to extract data because of the small number of articles. The primary researcher read and re-read the data to familiarise themselves with the data and develop an initial coding framework. Co-researchers then independently reviewed the initial codes. Adjustments to coding and themes continued through the regular revisiting of the data, with consensus established by review and discussion.

## Results

### Primary theme: Acting as a safety net

**Figure 2.**
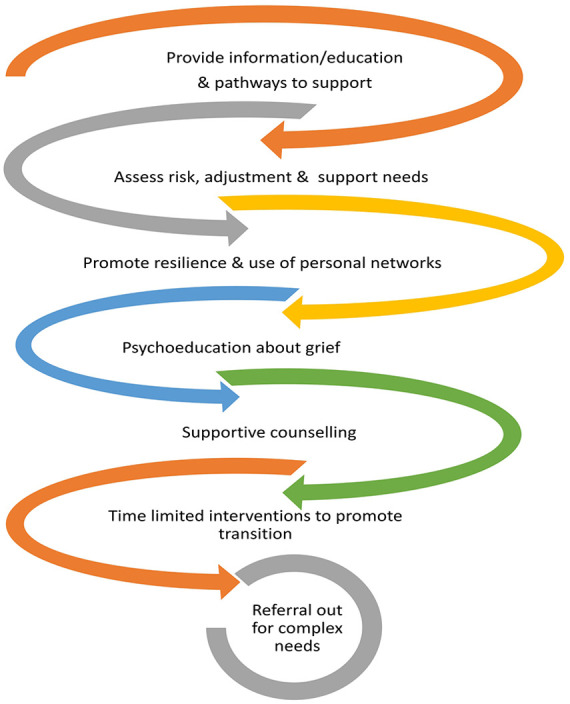
Palliative bereavement services acting as a safety net through service-led contact and supportive interventions.

We identified the primary purpose of service-initiated contact was to ‘act as a safety net’ with important elements shown in Figure 2 that were undertaken by bereavement services. Authors used a range of terms to describe the process of service-initiated bereavement contact and the provision of grief interventions. Service-initiated contact and intervention were perceived to provide a level of protection for the bereaved and aimed to increase grief literacy, self-awareness and coping. The findings are indicative of a continuum of care following the patient’s death to support people as they adjusted to their loss.

Two articles,^9,47^ referenced feedback from service users which provided some evidence of benefit. This was achieved through written information and various forms of grief-focussed interventions. Another article^48^ noted risk assessments targeting those with complex mental health needs. As reflected in Figure 2 and supported by the quotes in Table 2, most articles defined facilitating adjustment through a time-limited, supportive, active listening and educational approach to help make sense of the experience of grief. The safety net incorporated easy access to supportive grief counselling and/or group gatherings when desired by the bereaved individual, without incurring additional costs or the need for external referrals. Two sub-themes also emerged, comprising scheduled reviews and recognising service limits by referring to additional or specialist services. Indicative extracts from the articles are provided in Table 3.

**Table 3.** Sub-themes of safety through scheduled reviews and recognition of service limits.

References	Quotes from articles: Sub-theme: Safety net through scheduled reviews
Reid et al.^9^	‘A recurrent theme. . . with bereaved people was that. . . they. . . did not want to burden their families. . . [and] felt it was better to talk [outside of the family]’ (p. 431). ‘Helping them come to terms to with their grief and move on with their lives’ (p. 432).
Lattanzi^45^	‘Services are educational, preventative, and most importantly, supportive in nature. . .centred around specific goals. . .provide grieving family members an opportunity to review and reflect on their experience of caring . . . and their loss experience. Grief is. . .a complex time of need rather than an illness’ (p. 56). ‘An average of three to four visits are made to each individual/family. . . phone calls and notes are an important part of bereavement care’ (p. 59).
Harter Janson^46^	‘The level of individual support services that the bereaved client received was determined by his or her circumstances as assessed by the “Determinants of Grief” identified by Parkes. High risk survivors were assigned a . . .professional volunteer; [a nurse or social worker] other survivors were assigned a lay volunteer [not allied health trained]. [Support] varied according to the clients’ changing needs and wishes’ (p. 131).
Souter and Moore^47^	‘The execution of the program was identical for each risk category except for frequency of personal contacts. Primary goal . . . to offer reassurance and support’ . . .‘Volunteers . . . trained to help in facilitating . . .a gradual [adjustment] . . .and eventual reintegration of the bereaved into society’ (p. 34).
Bolotin^48^	‘Core values of justice and compassion, reflecting our . . .mission to service the most vulnerable. . .with the interns. . .the goal of providing phone outreach to 100 percent of our patients deaths within 30 days was rapidly achieved. . .’ (p. 47).
Khumalo and Maasdorp^51^	‘. . .comprehensive bereavement service through . . . social workers and trained volunteers, that provide or conduct individual counselling sessions, group therapy (children’s support, creative expression), widows support groups’ (p. 5). ‘The family is seen during contact bereavement visits and particular timings (first three months, six months. . ., one year. . .) are observed within the grief trajectory, although this is not formulaic in its application’ (p. 4).
References	Quotes from articles: Sub-theme: Recognising service limits: Referring for mental health treatment
Reid et al.^9^	[where there were] ‘. . .no readily available sources of support for people with complex bereavement problems. Long waiting lists for mental health services. . .’ (p. 437).
Lattanzi^45^	Individuals with ‘. . .pre-existing psychiatric problems or those in current treatment generally have greater needs than the follow-up could address’ (p. 56). ‘Outside referrals and resources are frequently included in the support of high risk individuals’ (p. 58). ‘Others who have concerns that are complicated by their grief are referred to local therapists’ (pp. 56–57).
Harter Janson^46^	‘. . .a person who was experiencing a complicated bereavement involving a pre-existing psychiatric disorder was referred to a mental health agency. . .’ (p. 130). ‘Volunteer [s] initiated referrals to other community resources, when necessary, to meet the client’s total needs’ (p. 131).
Souter and Moore^47^	Referrals to ‘. . .support resources were made if necessary. . .included. . .institutional resources. . .or for psychiatric problems. . .’ (p. 35).
Eastman et al.^50^	Referrals for further support aimed at ‘. . .effective referral pathways . . . to optimise care. . . and ease the burden of access for family members and . . .avoid service duplication’ (p. 961).
Ghesquiere et al.^52^	‘Additional services that can be offered by counsellors. . .outside referrals to additional mental health services as needed. . .’ (p. 371).

#### Sub-theme: Safety net through scheduled reviews

Several services described the provision of regular reviews as standard in their delivery of bereavement care. This form of *safety net* aimed to monitor adjustment through in-person reviews at set time points. Additional support options were also available, including group meetings, workshops, educational sessions and telephone contact. Services emphasised or indicated their continued relationship with the family throughout the first year of bereavement, typically as a process for regular review and monitoring of adjustment.^9,44–48,51^ Key descriptions of monitoring through scheduled reviews recognised grief as an adaptive process, involving complexity and challenge, and the need for services to be available. It was important that bereaved individuals were well informed that they could opt out if not wanting continued contact. Provision of scheduled reviews functioned as a *safety net* consisting of typically three to four face-to-face visits with supplementary telephone calls and various forms of group options made available. One article described a minimum of nine scheduled contacts in their programme.^46^

#### Sub-theme: Recognising service limits and referring for mental health services

The second sub-theme was the ability of services to know their limitations and ‘recognise the need for mental health’ interventions. Articles conveyed a process of referring to external services or additional support (where available). This was demonstrated through observations such as ‘outside referrals for mental health services’^46,47,52^ and ‘acknowledging existing therapy relationships’.^45,46^ One article notes ‘long waiting lists for mental health services’ though no further details are provided.^9^ When described, a distinction was typically made between referring for mental health or psychiatric services and the role of grief counselling. Differing locations for palliative services (such as the United Kingdom, Zimbabwe and the United States) influenced several factors. Access to mental health treatment options, long waiting times and availability of support, and a need to be covered by insurance, were considerations noted. As reflected in Figure 2 and supported by the quotes in Tables 2 and 3, articles described their role as time-limited, supportive contact to assess risk, monitor and facilitate adjustment and the provision of grief counselling. No service identified their primary purpose as therapy for prolonged grief. There was a recognition that bereavement services needed to be mindful of not creating dependence, as the purpose was to support people through a time of crisis as they transitioned and learnt to live with their loss.^9,45–48^

All articles highlighted the importance of a responsive approach to tailor support for individual needs and desires or the need for self-referral as a way of respecting individual resilience and autonomy. Six articles provided risk assessment information^45–47,49–50,52^ with the primary components of assessment outlined in Table 4. Where in-person contact occurred with the bereaved individual, a review of how they were responding to their grief would be attempted/completed as the primary risk assessment. A heightened risk of problems in adjusting to bereavement was indicated by experiencing concerning physical, psychological, emotional and spiritual symptoms, including anxiety or depression, suicidal ideation, overt anger, unintended weight changes, sleep disturbances, absence of family or social support, overwhelming stress and/or sense of hopelessness.

**Table 4.** Summary of indicators of concern when undertaking risk assessments.

Indicators considered when completing risk assessments included:	
• Quality of support system	• Multiple losses
• Family conflict	• Previous/current mental health history
• Financial or legal problems	• Issues related to patient’s death
• Emotional distress	• Functional impairment in daily living
• Coping strategies used	• Symptoms of anxiety/depression or early signs of prolonged grief (attachment style)

Informing the bereaved caregiver of options, providing a pathway to link into those options and making grief interventions accessible were seen as important. Authors wrote of helping family members to transition through an emotional and complex experience. Generally, the authors described the bereavement intervention model as a supportive process. They detailed grief interventions as available to bereaved people for a limited time to aid adjustment. Options consisted of telephone calls, group sessions, informative mailings, memorial gatherings and face-to-face grief counselling. No articles described that bereavement contact was providing therapy or treatment for prolonged grief. This was not unexpected, as it fits with the usual 12–13 month timeframe that palliative bereavement services typically limit support to.^6,7,10^ Bereavement support was available for differing lengths of time, ranging from a few weeks to 13 months. By contrast, prolonged grief is thought to be more reliably diagnosable from 12 to 18 months, as described in the *Diagnostic and Statistical Manual*, version 5 (DSM-5).^54,55^

Service-led bereavement contact aimed to connect with, complete a review and assessment of needs (when in-person) and inform people about available services and supports. Active listening and support to understand and manage the understanding of the challenges faced were key features of service aims. All services described the role of being there to support and guide and to provide grief counselling or other forms of support if needed. Acting as a safety net was achieved through actions including assessment, time-limited intervention and/or completing external referrals. One service limited their support to a few weeks following a patient’s death^51^ while others offered a continued relationship if required, through regular reviews during the first 12–13 months.^45–49^ Completion of risk assessment was a key component of several service models, with most defining the literature that informed the assessment.^45–49,51,52^ Provision of psycho-education about grief to assist in understanding grief experiences was claimed to promote personal resilience. There was a focus on building individual, family and community capacity in responding to the experience of grief. However, no articles provided a robust evidence base for their model of care which is an area worthy of much greater attention in future research.

## Discussion

Characteristics of palliative bereavement services have previously been described in several research articles.^3–10,37,56–59^ There is a surprising lack of research and detailed information relating to the bereavement practices of palliative care, given the needs in the community and increasing interest in policy and standards. This study has allowed us to identify common themes in the literature included. Findings also provide insight into some long-standing and current models of palliative bereavement services.

### What this study adds

Though only nine articles met the scoping review criteria, original pertinent data were extracted. The results showed the primary purpose and aim was for bereavement services to act as a safety net (see Figure 2). Sub-themes reiterate how bereavement services function as a safety net while knowing their limitations and the need to facilitate reconnection with personal networks. Support was directed at guiding and evaluating how the bereaved were adapting to the person’s death and to facilitating adjustment when indicated through grief-focussed interventions. Reviewed articles articulated the recognition of bereaved caregivers requiring time to adjust, the need to provide information, the benefit of connection with others and the encouraging use of the bereaved person’s natural network. In addition, most articles noted their time-limited, open-access approach to service provision and the necessity of time passing to allow grief to become integrated.

As such, the bereavement services can be seen to meet a need that may facilitate transition, and support a structured completion of the relationships with the palliative service. They offer a safety net to capture the more complex needs of those who are bereaved by an expected death within a palliative care service. This approach does not compete with the more purposeful interventions to address complicated grief, nor does it compete with the natural community networks that may wrap around the bereaved in ideal circumstances. Indeed, such contact can be seen to complement these strategies by normalising common grief experiences and the availability of community support resources, as well as prompting awareness of more formal pathways to respond to prolonged grief. The scoping review findings resonate with results from an unpublished companion study, a cross-sectional study of a specialist palliative bereavement service.^43^ In this 2017 cross-sectional study participants valued knowing the bereavement service was available and that this knowledge was helpful and reassuring, whether they made use of it or not.

The findings from our scoping review reflect how palliative bereavement services work alongside or in conjunction with suggested public health and tiered models^22,23,60^ and the *National Institute for Clinical Excellence*,^61^ approach to bereavement support. Our data indicates palliative bereavement services are an example of the suggested 3-tier or 4-tier models of bereavement support and are not replacing existing community networks.^22,23,60,61^ Services aimed to provide psycho-education about typical grief responses, to help normalise and self-assess grief reactions was a primary function (tiers 1–2) and provide grief-specific interventions (tiers 2–3) when required. Some articles acknowledged more complex mental health concerns could be present and, when noted, the focus was on supporting the bereaved person to receive appropriate treatment (tiers 3–4) if available. Lack of access to mental health services was recognised as a problem and highlights a limitation of bereavement services. A common philosophy and purpose were to develop personal and community capacity, through increasing grief literacy and supporting bereaved caregivers to build confidence in their own coping. Findings reflect a continuum of care and are particularly important for bereaved caregivers who can lose contact with their personal support networks.

There was evidence that bereavement services recognised some caregivers may form close bonds with palliative staff and can experience challenges in reconnecting to their broader support networks.^46,47,49^ Each service intended to provide an open model of access,^6^ grief support, guidance and information during the first months of bereavement. This was a way of facilitating the transition from caregiving *and* reconnecting to everyday life while coming to terms with the loss. There was a general sense of knowing service limits and ability to identify mental health concerns, demonstrated by acknowledging referral out to mental health services where able to.^9,45–47,52^ That said, it is critical to keep in mind the varying contexts of specialist palliative care and hospice services depending on their location and the influence of political and health systems, as there are implications for the provision of and access to bereavement support and grief counselling. While there were descriptions of support being provided to bereaved individuals until they felt able to manage on their own, discontinued support or required referral to more formalised interventions, we did not find any details on formally assessing outcomes following interventions.

### Limitations of the study

The primary aim of this scoping review was to examine how palliative and hospice bereavement services described their purpose and service models. Though the examined articles provide more information as to their model than are usually available from external surveys of palliative services^3,4,7,56–59^ limitations remain in the level of details. Regarding comprehensive detail about bereavement service intervention models, five of the publications answered most of the review questions.^45–47,50,52^ One article^9^ provided only limited insight into the actual practices of service-initiated bereavement contact and assessment procedures. Future research needs to obtain more precise and accurate information when examining the function and aims of palliative bereavement services; otherwise, there is a risk that imprecise information and reports may influence decisions made at the local service level. Similarly, poor reporting of how services are configured and perceived in the policy and research setting limits the use of the available data. Further, how these findings relate to populations of bereaved people that are underserved by palliative care services and the groups of people who are not able to access palliative bereavement support, is uncertain, though may indicate a need for similar processes. Articles were limited to only English studies, only a small number of articles were retrieved, and then from almost four decades. This may limit how the findings can be applied.

### Strengths of the study

This scoping has delivered unique evidence of bereavement models having a common approach through ‘acting as a safety net’ to the newly bereaved. A formally structured scoping review method has ensured rigour in the identification and extraction of core findings. To facilitate a thorough examination of the information, an extraction template was developed to consistently record details regarding the described function and method of service-initiated bereavement contact. To enhance the rigour of this study, two independent co-reviewers (a Clinical Psychologist, and PhD Social Worker) supported the primary researcher in the initial screening of abstracts and titles, full review of the included articles and thematic analysis. Themes were initially developed internally and then checked against two senior researchers (DC, JT) for their relevance and face validity. Review of the findings against public health and service bereavement approaches as well as a companion study further enable the value of the scoping review findings to be cross-checked.

### Implications for service delivery

For palliative bereavement services to focus their support model on acting as a safety net, which may also involve scheduled reviews for a time to monitor and facilitate positive adjustment makes intuitive sense as some bereaved caregivers are reported to be at heightened risk of prolonged grief^17,62–65^ often experience a decline in general health and experience other significant stressors.^11–20^ Service-initiated contact is important, and a minimum ‘recommended’ component of palliative care services in the immediate time following a patient’s death.^21^ Using standardised and validated methods of review would improve the evidence base for bereavement services.^17,27,65,66^ The provision of information on common grief reactions and promotion of self-determination in accessing grief counselling can be viewed as increasing grief literacy. Following individual preferences, tailoring support through regular reviews of adjustment and promotion of self-referral was evident. Analysis of service descriptions reflected the use of support when desired or indicated though did not suggest that blanket interventions were occurring. Nonetheless, the literature also notes there are some bereaved who find it difficult to accept support, or who do not recognise the need for intervention as they see their struggle as normal.^31^ Consequently, scheduled in-person reviews may be the best form of safety net to support assessment of individual needs and adjustment.

In addition, recognising that service-led contact, referrals and/or grief-focussed interventions provide a safety net can inspire other services to establish their own service-initiated contact processes if none already exist. While it is well known that not all bereaved individuals require grief counselling, there is a recognised need to provide information and support in the early period following a death. Specialist palliative services have a higher standard to meet regarding supporting grieving individuals due to the bonds that are formed with staff during end-of-life care in anticipated deaths. At minimum, specialist palliative services should be guiding bereaved family members to understand grief, how it may impact their functioning, and how support can be accessed.

### Implications for future research

Our scoping review provides a starting point in articulating the purpose of palliative bereavement service models, though much more is required. Firstly, there is a need for present-day palliative bereavement services to clearly articulate the purpose of their service-initiated contact and how they assess need for grief counselling. This requires bereavement services to be clear about their purpose and intent. Such clarity could be shared through publications and when surveys are conducted. Without such clarity in describing the aim and intent of service-initiated contact, assessment and grief counselling interventions, concerns like the one expressed by some literature^22–28^ will continue due to lack of detailed information. Aligning with this scoping review, an insightful description can be found in Kobel et al.^6^ survey of Australian palliative bereavement services which appears to be in-step with the published service descriptions included in this scoping review. They acknowledged that ‘access for all’ (which reflects our concept of a safety net) differs from ‘provision to all’. Secondly, more research is needed to examine how current palliative bereavement services define their work, to help validate the investment made, all be it a typically small investment. Bereavement services may in fact view their primary purpose as providing information and pathways to support, thus acting as a safety net, though they have yet to adopt this term. Services may also define an equally important purpose of completing an assessment of needs with the bereaved person and providing time-limited grief counselling when indicated, available and requested. Palliative bereavement services may find a natural ally in the compassionate community movement, where they could form a partnership in helping families transition from formal health services back into community networks. Adopting this process would be like a recently proposed model,^67^ with a similar approach described in one review article.^48^

In the future, policy approaches and funding bodies may need to measure models of bereavement care from specialist palliative services. Examining bereavement service models and activity against healthcare accreditation standards, for example, will further add to emerging evidence. Obtaining such evidence about current service models and how this is experienced by the bereaved individual should be considered a priority, due to the known risk of declines in health and potential for loss of life. Inclusive research about service models, meaning research undertaken with providers of bereavement support, will improve the understanding of what palliative bereavement services offer. Research in conjunction with bereavement service providers and those it is aimed at plays a key role in obtaining much-needed clarity of models of care, their effectiveness and impact. Co-constructed research will identify what bereaved individuals seek and how services meet or fail to meet that demand. Consideration also needs to be given to how current models of bereavement care best meet varying cultural needs, across different age groups and in various settings. Research on access to bereavement support is generally from high-income countries, where personal choice and responsibility is emphasised and highly valued, and personal income and public health access may influence models of care. We acknowledge that as our research was conducted before the COVID-19 pandemic literature emerged, some palliative care services may have revised their model of bereavement support. Recent literature indicates an increased focus on bereavement support.^68,69^

## Conclusion

Our findings show palliative bereavement services aim to provide support according to identified and expressed needs. The findings of this scoping review indicate bereavement service models have an overall common purpose when considered as an accessible and recognisable safety net. Such service models can guide newly bereaved individuals to understand their responses and support decision-making about the need for formal interventions for complex needs. Further, this study offers a start to shared terminology regarding service-initiated bereavement contact and grief counselling processes. If the literature is to move on from its concerns about specialist palliative bereavement service models, then bereavement services have some work to do. Further, in the face of increasing pressure on resources, palliative bereavement services need to articulate their approach clearly to be better understood and specific in how their practices meet national standards to better understand their purpose and impact.

## Supplemental Material

sj-docx-1-pcr-10.1177_26323524251326947 – Supplemental material for What functions do palliative care bereavement services deliver? A scoping review Supplemental material, sj-docx-1-pcr-10.1177_26323524251326947 for What functions do palliative care bereavement services deliver? A scoping review by Kathleen E. Jurgens, David C. Currow and Jennifer Tieman in Palliative Care and Social Practice

sj-docx-2-pcr-10.1177_26323524251326947 – Supplemental material for What functions do palliative care bereavement services deliver? A scoping review Supplemental material, sj-docx-2-pcr-10.1177_26323524251326947 for What functions do palliative care bereavement services deliver? A scoping review by Kathleen E. Jurgens, David C. Currow and Jennifer Tieman in Palliative Care and Social Practice

sj-docx-3-pcr-10.1177_26323524251326947 – Supplemental material for What functions do palliative care bereavement services deliver? A scoping review Supplemental material, sj-docx-3-pcr-10.1177_26323524251326947 for What functions do palliative care bereavement services deliver? A scoping review by Kathleen E. Jurgens, David C. Currow and Jennifer Tieman in Palliative Care and Social Practice

## Declarations

**Ethics approval and consent to participate:** This article does not contain any studies with human or animal participants.

**Consent for publication:** Not applicable.

**Availability of data and materials:** Due to the nature of this review, data has been shared throughout.

## References

[1] World Health Assembly, 67. Strengthening of palliative care as a component of comprehensive care throughout the life course. Sixty-seventh World Health Assembly, 2014, pp. 9–14. https://iris.who.int/handle/10665/162863 (last accessed 10 March 2025).

[2] Australian Government, Palliative Care Australia. National palliative care standards. 5.1 ed., https://palliativecare.org.au/national-palliative-care-standards (2024, last accessed 10 March 2025).

[3] Abbott JA O’Connor M Payne S. An Australian survey of palliative care and hospice bereavement services. Aust J Cancer Nurs 2008; 9(2): 12–17.

[bibr4-26323524251326947] Field D Reid D Payne S , et al. Survey of UK hospice and specialist palliative care adult bereavement services. Intern J Palliat Nurs 2004; 10(12): 569–576. 10.12968/ijpn.2004.10.12.17280 15750516

[bibr5-26323524251326947] Foliart E Clausen M Siljestrom C. Bereavement practices among California hospices: results of a statewide survey. Death Stud 2001; 25(5): 461–467. 11806414 10.1080/07481180125792

[bibr6-26323524251326947] Kobel C Morris D Thompson C , et al. Bereavement support in palliative care: a national survey of Australian services. J Palliat Med 2019; 22(8): 933–938. 30794018 10.1089/jpm.2018.0502

[bibr7-26323524251326947] Mather MA Good PD Cavenagh JD , et al. Survey of bereavement support provided by Australian palliative care services. Med J Aust 2008; 188(4): 228–230. 18279130 10.5694/j.1326-5377.2008.tb01590.x

[bibr8-26323524251326947] Arriaza P Martin SS Csikai EL. An assessment of hospice bereavement programs for Hispanics. J Soc Work End Life Palliat Care 2011; 7(2-3): 121–138. 21895433 10.1080/15524256.2011.593151

[bibr9-26323524251326947] *Reid D Field D Payne S , et al. Adult bereavement in five English hospices: types of support. Int J Palliat Nurs 2006; 12(9): 430–437. 17077802 10.12968/ijpn.2006.12.9.21871

[10] Roberts A McGilloway S. The nature and use of bereavement support services in a hospice setting. Palliat Med 2008; 22(5): 612–625. 18612027 10.1177/0269216308090071

[11] Carey IM Shah SM DeWilde S , et al. Increased risk of acute cardiovascular events after partner bereavement: a matched cohort study. JAMA Intern Med 2014; 174(4): 598–605. 24566983 10.1001/jamainternmed.2013.14558

[bibr12-26323524251326947] Jones MP Bartrop RW Forcier L , et al. The long-term impact of bereavement upon spouse health: a 10-year follow-up. Acta Neuropsychiatr 2010; 22(5): 212–217. 26952830 10.1111/j.1601-5215.2010.00482.x

[bibr13-26323524251326947] Stroebe M Schut H Stroebe W. Health outcomes of bereavement. Lancet 2007; 370(9603): 1960–1973. 18068517 10.1016/S0140-6736(07)61816-9

[bibr14-26323524251326947] Aoun SM Kissane DW Cafarella PA , et al. Grief, depression, and anxiety in bereaved caregivers of people with motor neurone disease: a population-based national study. Amyotrophic Lateral Scler Frontotemporal Degener 2020; 21(7–8): 593–605. 10.1080/21678421.2020.1790610 32668960

[bibr15-26323524251326947] Moriarty J Maguire A O’Reilly D , et al. Bereavement after informal caregiving: assessing mental health burden using linked population data. Am J Public Health 2015; 105(8): 1630–1637. 26066918 10.2105/AJPH.2015.302597 PMC4504303

[bibr16-26323524251326947] Zordan RD Bell ML Price M , et al. Long-term prevalence and predictors of prolonged grief disorder amongst bereaved cancer caregivers: a cohort study. Palliat Support Care 2019; 17(5): 507–514. 30767818 10.1017/S1478951518001013

[bibr17-26323524251326947] McCabe PJ Bor W. Bereavement is different: a multinational bereavement symptom model validation. Psychiatry Res 2021; 300: 113926. 33872854 10.1016/j.psychres.2021.113926

[bibr18-26323524251326947] Thimm J Kristoffersen AE Ringberg U. The prevalence of severe grief reactions after bereavement and their associations with mental health, physical health, and health service utilization: a population-based study. Eur J Psychotraumatol 2020; 11(1): 1844440. 33408813 10.1080/20008198.2020.1844440 PMC7748058

[bibr19-26323524251326947] Shimizu Y Masukawa K Aoyama M , et al. The impact of stressful life events after bereavement: a nationwide cross-sectional survey. J Pain Symptom manage 2023; 65(4): 273–284. 36584737 10.1016/j.jpainsymman.2022.12.012

[20] Lancaster H Johnson T. Losing a partner: the varying financial and practical impacts of bereavement in different sociodemographic groups. BMJ Support Palliat Care 2020; 10(2): e17. 10.1136/bmjspcare-2016-001215 28450441

[21] Hudson P Hall C Boughey A , et al. Bereavement support standards and bereavement care pathway for quality palliative care. Palliat Support Care 2018; 16(4): 375–387. 28701233 10.1017/S1478951517000451

[22] Aoun S Breen L O’Connor M , et al. A public health approach to bereavement support services in palliative care. Aust N Z J Public Health 2012; 36(1): 14–16. 22313700 10.1111/j.1753-6405.2012.00825.x

[bibr23-26323524251326947] Aoun SM Breen LJ Howting DA , et al. Who needs bereavement support? A population based survey of bereavement risk and support need. PLoS One 2015; 10(3): e0121101. 10.1371/journal.pone.0121101 PMC4374848 25811912

[bibr24-26323524251326947] Aoun S Slatyer S Deas K , et al. Family caregiver participation in palliative care research: challenging the myth. J Pain Symptom Manage 2017; 53(5): 851–861. 28062338 10.1016/j.jpainsymman.2016.12.327

[bibr25-26323524251326947] Breen LJ Aoun SM O’Connor M , et al. Bridging the gaps in palliative care bereavement support: an international perspective. Death Stud 2014; 38(1): 54–61. 24521046 10.1080/07481187.2012.725451

[bibr26-26323524251326947] Rumbold B Aoun S. Bereavement and palliative care: a public health perspective. Prog Palliat Care 2014; 22(3): 131–135.

[bibr27-26323524251326947] Sealey M O’Connor M Aoun SM , et al. Exploring barriers to assessment of bereavement risk in palliative care: perspectives of key stakeholders. BMC Palliat Care 2015; 14: 1–2. 26466576 10.1186/s12904-015-0046-7 PMC4606550

[28] Schut H. Grief counselling efficacy: have we learned enough? Bereavement Care 2010; 29(1): 8–9.

[29] Bergman EJ Haley WE Small BJ. Who uses bereavement services? An examination of service use by bereaved dementia caregivers. Aging Ment Health 2011; 15(4): 531–540. 21500020 10.1080/13607863.2010.543661

[30] Cherlin EJ Barry CL Prigerson HG , et al. Bereavement services for family caregivers: how often used, why, and why not. J Palliat Med 2007; 10(1): 148–158. 17298263 10.1089/jpm.2006.0108

[31] Lichtenthal WG. Supporting the bereaved in greatest need: we can do better. Palliat Support Care 2018; 16(4): 371–374. 30226127 10.1017/S1478951518000585

[32] Roberts K Holland J Prigerson HG , et al. Development of the Bereavement Risk Inventory and Screening Questionnaire (BRISQ): item generation and expert panel feedback. Palliat Support Care 2017; 15(1): 57–66. 27516152 10.1017/S1478951516000626 PMC5296244

[33] Harrop E Morgan F Longo M , et al. The impacts and effectiveness of support for people bereaved through advanced illness: a systematic review and thematic synthesis. Palliat Med 2020; 34(7): 871–888. 32419630 10.1177/0269216320920533 PMC7341024

[34] Hasson F Spence A Waldron M , et al. Experiences and needs of bereaved carers during palliative and end-of-life care for people with chronic obstructive pulmonary disease. J Palliat Care 2009; 25(3): 157–163. 19824276

[35] Harrop E Morgan F Byrne A , et al. ‘It still haunts me whether we did the right thing’: a qualitative analysis of free text survey data on the bereavement experiences and support needs of family caregivers. BMC Palliat Care 2016; 15: 1–8. 27825330 10.1186/s12904-016-0165-9 PMC5101847

[36] Vierhout M Varenbut J Amos E , et al. Loss of relationship: a qualitative study of families and healthcare providers after patient death and home-based palliative care ends. Ann Palliat Med 2019; 8(2): 13039–13139. 10.21037/apm.2019.03.01 30943737

[37] O’Connor M Abbott JA Payne S , et al. A comparison of bereavement services provided in hospice and palliative care settings in Australia, the UK and the USA. Prog Palliat Care 2009; 17(2): 69–74.

[38] Peters MD Marnie C Tricco AC , et al. Updated methodological guidance for the conduct of scoping reviews. JBI Evid Synth 2020; 18(10): 2119–2126. 33038124 10.11124/JBIES-20-00167

[bibr39-26323524251326947] Munn Z Peters MD Stern C , et al. Systematic review or scoping review? Guidance for authors when choosing between a systematic or scoping review approach. BMC Med Res Method 2018; 18: 1–7. 10.1186/s12874-018-0611-x PMC6245623 30453902

[bibr40-26323524251326947] Arksey H O’Malley L. Scoping studies: towards a methodological framework. Intern J Soc Res Method 2005; 8(1): 19–32.

[41] Tricco AC Lillie E Zarin W , et al. PRISMA Extension for Scoping Reviews (PRISMAScR): checklist and explanation. Ann Intern Med 2018; 169: 467–473. 30178033 10.7326/M18-0850

[42] Glenton C Lewin S Downe S , et al. Cochrane Effective Practice and Organisation of Care (EPOC) qualitative evidence syntheses, differences from reviews of intervention effectiveness and implications for guidance. Int J Qual Methods 2022; 21: 16094069211061950.

[43] Jurgens K. A cross-sectional survey of bereaved caregivers’ perspectives on preparedness, moving forward and impact of support from a palliative care bereavement service, including a scoping review of bereavement service descriptions. Unpublished PhD Thesis, Flinders University, South Australia, 2023.

[44] Tieman JJ Abernethy AP Fazekas BS , et al. CareSearch: finding and evaluating Australia’s missing palliative care literature. BMC Palliat Care 2005; 4: 1–9. 16083513 10.1186/1472-684X-4-4 PMC1190188

[bibr45-26323524251326947] *Lattanzi ME. Hospice bereavement services: creating networks of support. Family Commun Health 1982; 5(3): 54–63. 10.1097/00003727-198211000-00009 10258221

[bibr46-26323524251326947] *Harter Janson MA . A comprehensive bereavement program. QRB Qual Rev Bull 1986; 12(4): 130–135. 3086792 10.1016/s0097-5990(16)30027-6

[bibr47-26323524251326947] *Souter SJ Moore TE. A bereavement support program for survivors of cancer deaths: A description and evaluation. Omega J Death Dying 1990; 20(1): 31–43.

[bibr48-26323524251326947] *Bolotin SM. Bereavement program develops initiative with universities for win-win partnership: obtains record-breaking community outcomes. Caring 2008; 27(11): 46–49. 19068877

[49] *Agnew A Manktelow R Haynes T , et al. Bereavement assessment practice in hospice settings: Challenges for palliative care social workers. Br J Soc Work 2011; 41(1): 111–130.

[50] *Eastman P Le B Pharaoh A. The establishment and initial outcomes of a palliative care bereavement service. Palliat Med 2012; 26(7): 961–962. 22995826 10.1177/0269216312438931

[51] *Khumalo T Maasdorp V. The Island Hospice model of palliative care. Ecancermedicalscience 2016; 10: 654. 27563349 10.3332/ecancer.2016.654 PMC4970623

[52] *Ghesquiere A Bagaajav A Metzendorf M , et al. Hospice bereavement service delivery to family members and friends with bereavement-related mental health symptoms. Am J Hosp Palliat Med 2019; 36(5): 370–378. 10.1177/1049909118812025 PMC6486888 30428680

[53] Noyes J Lewin S. Chapter 5: Extracting qualitative evidence. In: Noyes J Booth A Hannes K , et al. (eds.) Supplementary guidance for inclusion of qualitative research in cochrane systematic reviews of interventions. Version 1 (updated August 2011). Cochrane Collaboration Qualitative Methods Group. 2011, pp. 1–24. http://cqrmg.cochrane.org/supplemental-handbook-guidance (last accessed 10 March 2025).

[54] Prigerson HG Boelen PA Xu J , et al. Validation of the new DSM-5-TR criteria for prolonged grief disorder and the PG-13-Revised (PG-13-R) scale. World Psychiatry 2021; 20(1): 96–106. 33432758 10.1002/wps.20823 PMC7801836

[55] Szuhany KL Malgaroli M Miron CD , et al. Prolonged grief disorder: course, diagnosis, assessment, and treatment. Focus 2021; 19(2): 161–172. 34690579 10.1176/appi.focus.20200052 PMC8475918

[56] Barry CL Carlson MD Thompson JW , et al. Caring for grieving family members: results from a national hospice survey. Med Care 2012; 50(7): 578–584. 22310561 10.1097/MLR.0b013e318248661d PMC3374048

[57] Demmer C. A national survey of hospice bereavement services. OMEGA J Death Dying 2003; 47(4): 327–341.

[58] Matsushima T Akabayashi A Nishitateno K. The current status of bereavement follow-up in hospice and palliative care in Japan. Palliat Med 2002; 16(2): 151–158. 11969146 10.1191/0269216302pm522oa

[bibr59-26323524251326947] Guldin MB Murphy I Keegan O , et al. Bereavement care provision in Europe: a survey by the EAPC bereavement care taskforce. Eur J Palliat Care 2015; 22(4): 185–189.

[bibr60-26323524251326947] The Irish Hospice Foundation. Adult bereavement care pyramid – a national framework [Online]. Dublin: The Irish Hospice Foundation, https://hospicefoundation.ie/wp-content/uploads/2021/02/Adult-Bereavement-Care-Framework-Pyramid-Booklet.pdf (2020, last accessed 10 March 2025).

[61] National Institute for Clinical Excellence. Guidance on cancer services: improving supportive and palliative care for adults with cancer. London: National Institute for Clinical Excellence, 2004. https://www.nice.org.uk/guidance/csg4 (last accessed 10 March 2025).

[62] Givens JL Prigerson HG Kiely DK , et al. Grief among family members of nursing home residents with advanced dementia. Am J Geriatr Psychiatry 2011; 19(6): 543–550. 21606897 10.1097/JGP.0b013e31820dcbe0 PMC3101368

[bibr63-26323524251326947] Schulz R Boerner K Shear K , et al. Predictors of complicated grief among dementia caregivers: a prospective study of bereavement. Am J Geriatr Psychiatry 2006; 14(8): 650–658. 16861369 10.1097/01.JGP.0000203178.44894.db

[bibr64-26323524251326947] Trevino KM Litz B Papa A , et al. Bereavement challenges and their relationship to physical and psychological adjustment to loss. J Palliat Med 2018; 21(4): 479–488. 29182478 10.1089/jpm.2017.0386 PMC5867503

[65] Agnew A Manktelow R Taylor BJ , et al. Bereavement needs assessment in specialist palliative care: a review of the literature. Palliat Med 2010; 24(1): 46–59. 19762368 10.1177/0269216309107013

[66] Newsom C Schut H Stroebe MS , et al. Initial validation of a comprehensive assessment instrument for bereavement-related grief symptoms and risk of complications: the Indicator of Bereavement Adaptation—Cruse Scotland (IBACS). PLoS One 2016; 11(10): e0164005. 10.1371/journal.pone.0164005 PMC5065141 27741246

[67] Lichtenthal WG Roberts KE Donovan LA , et al. Investing in bereavement care as a public health priority. Lancet Public Health 2024; 9(4): e270–e274. 10.1016/S2468-2667(24)00030-6 PMC11110717 38492580

[68] Mateus MJ Simões L Ali AM , et al. Family experiences of loss and bereavement in palliative care units during the COVID-19 pandemic: an interpretative phenomenological study. Healthcare 2024; 12: 1763. 39273788 10.3390/healthcare12171763 PMC11395245

[69] Harrop E Goss S Farnell D , et al. Support needs and barriers to accessing support: Baseline results of a mixed-methods national survey of people bereaved during the COVID-19 pandemic. Palliat Med 2021; 35(10): 1985–1997. 34676792 10.1177/02692163211043372 PMC8637353

