# Supplementary material for: What functions do palliative care bereavement services deliver? A scoping review
Source: Palliat Care Soc Pract. 2025 Mar 22;19:26323524251326947. doi: 10.1177/26323524251326947 (PMC11946289; doi:10.1177/26323524251326947)
Supplement: sj-docx-2-pcr-10.1177_26323524251326947 – Supplemental material for What functions do palliative care bereavement services deliver? A scoping review [file sj-docx-2-pcr-10.1177_26323524251326947.docx]

Supplement 2: Search Terms Medline February 2020 and December 2024

| **#** | **Searches** | **Results** |
| --- | --- | --- |
| 1 | bereavement/ or grief/ | 13021 |
| 2 | (bereave* or grief* or grieving or mourn*).tw,kw. | 13867 |
| 3 | ((loss or death or "passed away") adj2 (family* or families or spous* or parent or parents or father* or mother* or friend or friends or husband* or wife or wives or partner or partners or neighbour* or neighbor* or "significant other*" or offspring or adolescent or sibling* or "loved one" or adult)).tw,kf. | 5134 |
| 4 | exp Bereavement/ | 13026 |
| 5 | exp Terminal Care/ | 50361 |
| 6 | exp Hospices/ | 4954 |
| 7 | exp Palliative Care/ | 52810 |
| 8 | exp Terminally Ill/ | 6423 |
| 9 | (palliat or palliata or palliate or palliated or palliatedwithout or palliates or palliatice or palliatieve or palliatifs* or palliating or palliationdagger or palliations or palliative* or palliative or palliative* or palliativealliance or palliativecare or palliativedrugs or palliativeinrichtungen or palliativelly or palliatively or palliativer or palliatives or palliatives* or palliativesurgery or palliativist or palliativists or palliativmedizin or palliativpatienten or palliativpflege or palliativsituation or palliativt or palliativversorgung or palliativwissenstest or palliativwissenstests or palliaton or palliator or palliatory or palliatum or palliates or palliatusindecorus or palliatuve or hospice or hospice' or hospice's or hospicecare or hospicelike or hospicelink or hospicelink* or hospicells or hospicells or hospicentric or hospices or hospices* or hospicewide).tw. | 70557 |
| 10 | or/1-9 | 149790 |
| 11 | (paediatric* or pediatric* or miscarriage* or stillbirth or neonat* or perinatal or ((death or loss) adj3 (child or baby or pregnancy or infant))).ti,ab,kf. | 676023 |
| 12 | (suicide or ((death or loss) adj3 (sudden or accidental))).ti,ab,kf. | 110076 |
| 13 | or/11-12 | 777241 |
| 14 | 10 not 13 | 137515 |
| 15 | caregivers/ | 35148 |
| 16 | family/ or exp family characteristics/ or family relations/ or intergenerational relations/ or friends/ or exp parents/ or spouses/ or visitors to patients/ or family therapy/ or family health/ | 277392 |
| 17 | (family* or families or spous* or parent or parents or father* or mother* or friend or friends or husband* or wife or wives or partner or partners or neighbour or neighbours or "significant other*" or offspring or sibling* or "loved one*").tw,kf. | 1531044 |
| 18 | next of kin.tw. | 1399 |
| 19 | or/15-18 | 1634601 |
| 20 | (outreach or "follow up" or "follow-up" or contact or "early intervent*" or "condolence letter" or "post-death meeting" or debrief* or "remembrance event*" or "bereavement service" or "bereavement support" or "bereavement care").tw,kf. | 1254654 |
| 21 | 14 and 19 and 20 | 1672 |
