# Supplementary material for: What functions do palliative care bereavement services deliver? A scoping review
Source: Palliat Care Soc Pract. 2025 Mar 22;19:26323524251326947. doi: 10.1177/26323524251326947 (PMC11946289; doi:10.1177/26323524251326947)
Supplement: sj-docx-3-pcr-10.1177_26323524251326947 – Supplemental material for What functions do palliative care bereavement services deliver? A scoping review [file sj-docx-3-pcr-10.1177_26323524251326947.docx]

Supplement 3: Scoping Review Data Extraction Template

| **Article title** | **Setting, method of article/study, and service-initiated contact method** | **Risk screening, assessment of need with bereaved, purpose of service-initiated bereavement contact informing about and commencing support** |
| --- | --- | --- |
| Funding: | Service-initiated bereavement service contact method | Risk screen:  Assessment of need for bereavement support with bereaved caregiver:  Purpose:  Informing about support versus commencing: |
